# Dual-Energy CT Arthrography: Advanced Muscolo-Skelatal Applications in Clinical Practice

**DOI:** 10.3390/tomography9040117

**Published:** 2023-08-08

**Authors:** Giovanni Foti, Christian Booz, Giuseppe Mauro Buculo, Eugenio Oliboni, Chiara Longo, Paolo Avanzi, Antonio Campacci, Claudio Zorzi

**Affiliations:** 1 Department of Radiology, IRCCS Sacro Cuore Hospital, Via Don A. Sempreboni 10, 37042 Negrar, Verona, Italy; eugenio.oliboni@sacrocuore.it (E.O.); chiara.longo@sacrocuore.it (C.L.); 2 Department of Diagnostic and Interventional Radiology, Division of Experimental Imaging, University Hospital Frankfurt, 60596 Frankfurt am Main, Germany; boozchristian@gmail.com; 3 Department of Radiology, Messina University, 98122 Messina, Sicily, Italy; giuseppe.buculo@gmail.com; 4 Department of Orthopedic Surgery, IRCCS Sacro Cuore Hospital, 37042 Negrar, Verona, Italy; paolo.avanzi@sacrocuore.it (P.A.); antonio.campacci@sacrocuore.it (A.C.);

**Keywords:** CT, arthrography, dual-energy, shoulder, hip

## Abstract

This paper provides a comprehensive overview of the potential applications of dual-energy CT (DECT) in improving image quality and the diagnostic capabilities of CT arthrography (CTA) in clinical practice. The paper covers the use of virtual non-contrast (VNC) images, in which the injected contrast medium is subtracted from the articular cavity in order to better analyze 2D and 3D images of the bone. Moreover, virtual monoenergetic imaging (VMI) applications and their potential use for the reduction of metal artifacts and improving image contrast are reviewed. The role of virtual non-calcium (VNCa) in detecting bone marrow edema surrounding the imaged joint will be discussed. Furthermore, the role of iodine maps in enhancing the contrast between soft tissues, optimizing the visualization of contrast material, and distinguishing contrast material from calcifications is described. Finally, a case series including different joints is provided to underline the additional advantages of high-spatial-resolution dual-energy CT reconstructed images.

## 1. Introduction

Arthrography is the most efficient imaging tool employed to diagnose complex joint lesions preoperatively [1]. Arthrograms can show ligament, tendon, and cartilage issues in clear detail by distending the articular cavity with injected contrast material [2]. Magnetic resonance arthrography (MRA) represents the most reliable exam to assess both large and small articulations because of its intrinsic high contrast resolution for soft tissue imaging [3,4,5,6,7,8]. However, it is an expensive exam with a long acquisition time; in addition, some drawbacks such as metallic implants, claustrophobia, and long waiting times are increasing in daily practice.

Dual-energy CT (DECT) represents a relatively new technology capable of characterizing tissues and other materials (e.g., iodine) because of different attenuation values at different energy levels [9,10]. Dedicated applications and relative maps were proposed to identify gout and bone marrow edema (BME) and reduce artifacts around metal implants [11,12,13,14,15,16].

Multiple applications of DECT could be employed to improve the diagnostic capabilities of CT arthrography [17,18].

For example, DECT virtual non-contrast (VNC) images may be used to avoid the acquisition of non-contrast images, with a subsequent reduction in radiation dose to the patient [19,20]. VNC images are routinely employed for the assessment of abdominal parenchyma [21] and vascular imaging [22]. Moreover, VNC images from dual-energy CT arthrography (DECTA) of the shoulder with iodine removal have been proposed for the assessment of the glenoid morphology and for quantitative measurements of the glenoid area [19]. Similarly, 2D and 3D high-resolution CT bone images can be used for pre-operative planning in cases of femoro-acetabular impingement syndrome [20]. This clinical need for the identification and quantification of both cartilaginous and bone defects is of great importance for the surgeon, especially for those patients with shoulder dislocation. Additionally, 3D reconstruction, allowed by CT, is now often requested by orthopedic surgeons for hip, knee, ankle, and elbow surgery as well, because it is employed for surgical planning. In this scenario, CT could play a key role in representing a “one-stop one-shop” exam, allowing both intra-articular evaluation and 3D assessment of the involved joint.

Additionally, there are organizational issues, with an increasing demand for shoulder, wrist, and hip arthrography even in relatively older patients (for example, to demonstrate the presence of some specific lesions for insurance policy issues). In these cases, CT could successfully replace MRI, being quite accurate and reliable without the additional problem of radiation burden that concerns young patients.

In addition, DECT can be employed to identify BME in traumatic and non-traumatic settings [21,22,23,24,25,26,27,28,29,30,31], to detect signs of inflammation in cases of inflammatory arthritis [32,33] and to detect soft tissue lesions [34,35,36]. The presence of BME around the joint may help to clarify the diagnosis, especially in cases of trauma.

Moreover, virtual mono-energetic imaging (VMI) has been proposed to reduce artifacts around metallic components in operated patients [23,24] and also to optimize the visualization of contrast material injected into the articular cavity [19]. The dilution of contrast material may indeed vary depending on the concentration of contrast material employed and on the presence of pre-existing fluid within the articular cavity. Furthermore, DECT has been used for the identification of intra-articular loose bodies in shoulder CTA [10].

In addition, iodine maps were proposed for DECTA of the shoulder to optimize the visualization of the contrast material injected and improve accuracy compared to standard CTA [25]. Similarly, iodine maps could be employed to distinguish intra- or peri-articular calcifications from iodine, such as rotator cuff tears.

Finally, DECT is characterized by an intrinsic increase in soft-tissue contrast, such as in collagen imaging applications, that can be employed to enhance the visualization of ligaments and tendons around the imaged joints [34,35,36].

Although the availability and use of DECT scanners and techniques are increasing, access to these exams is still limited to referral centers.

Moreover, further technical development, such as photon-counting CT with a very high spatial resolution at a relative low dose of radiation, could further increase the potential benefits of CT arthrography in clinical practice, but there are no studies available yet.

The purpose of this narrative review is to assess and discuss the benefits and pitfalls of the DECTA application in clinical practice.

## 2. DECT Imaging Protocol and Post-Processing

DECTA examinations can be performed with any dual-energy or spectral CT scanner. In our clinical practice, we use a dual-source DECT scanner (Somatom^®^ Definition Force, Siemens Healthcare, Forchheim, Germany).

At our institution, the injection of contrast material is performed under ultrasound or radioscopic guidance with a 22-gauge spinal needle, according to the operator’s choice. We use a mixture of iodinated contrast material (Iopamiro 370, Iopamidolo, 3.7 g of iodine, Bracco Imaging, Milan, Italy) and saline (1:2 ratio).

For DECT, patients are positioned supine, with the target articulation in a neutral position. The scanning parameters were as follows: tube A at 80 kV and tube B at 150 kV with a tin filter. The tube current–time products for tubes A and B were 220 and 138 reference mAs, respectively. Automated attenuation-based tube current modulation should be employed to limit the radiation burden.

During post-processing, we achieve three datasets of images (thickness 0.75 mm; increment 0.6 mm): an 80-kVp set, a 150-kVp set, and a blended virtual 120 kVp set of images with a Br 64 kernel—osteo-window filter. Moreover, soft-tissue kernel (Qr32) 80-kVp and 150-kVp sets of images are reconstructed on an off-line workstation (SyngoVia^®^ VB20; Siemens, Erlangen, Germany). A three-material decomposition algorithm is applied, and multiple lookup tables are available. Applications are chosen according to the radiologist’s preference. VMI is employed to achieve a VNC dataset for bone measurements or to reduce artifacts in the case of metallic components. Iodine maps are used to enhance the visualization of subtle labral or tendon tears. Additionally, in the case of small articulations such as the wrist or elbow, 0.3 mm reconstructions are obtained to improve the visualization of tiny articular anatomic structures and subtle cartilage lesions. Finally, BME maps are obtained in cases of trauma to rule out or confirm the presence of BME around the imaged joint.

## 3. Virtual Non-Contrast (VNC) Images and Virtual Monoenergetic Imaging (VMI)

**Figure 1:**
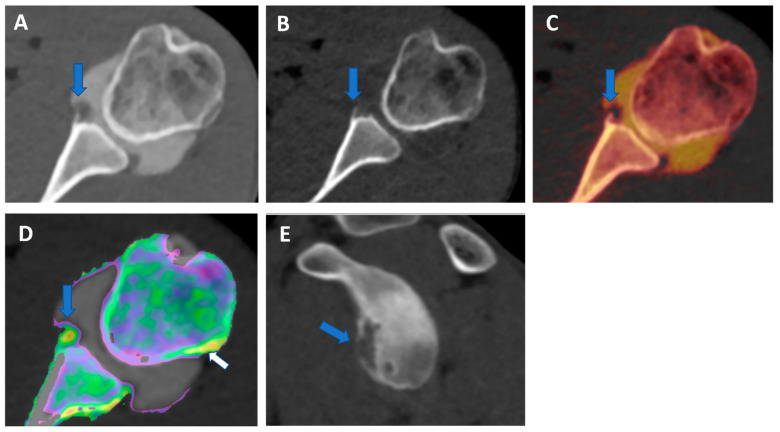
Recurrent anterior shoulder dislocation in a previously operated patient. By using multiple applications, DECTA can help in the diagnosis of anterior shoulder dislocation, potentially representing a “one-stop one-shop” procedure. A blended virtual 120 kVp axial CTA image (**A**) shows anterior labral and glenoid rim disruption (arrow). A VNC image on the axial (**B**) and sagittal (**E**) planes helps in the evaluation of bone morphology, allowing correct glenoid surface measurement (arrow). In the axial iodine map (**C**), it is possible to better evaluate the morphology of the anterior labrum (arrow). In the axial BME 2D superimposed image (**D**), it is possible to recognize edema of the anterior glenoid rim (blue arrow) and subtle edema of the posterior aspect of the humeral head (white arrow), which is consistent with recent recurrent dislocation.

**Figure 2:**
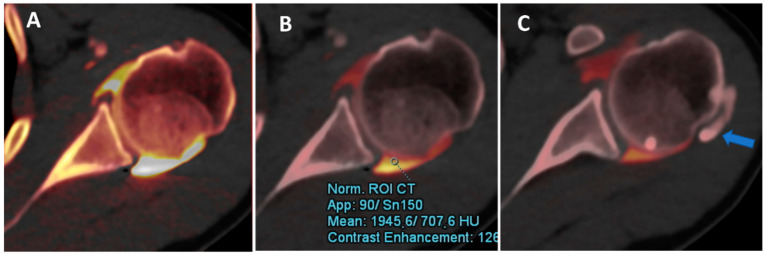
DECTA iodine map images. The iodine map from DECT can be used to optimize the dilution and visualization of contrast material injected and to differentiate contrast from calcifications. In this case, the axial iodine map DECTA image (**A**) shows a poorly diluted contrast, creating some artifacts near the posterior labrum. By normalizing contrast material to the density of the less diluted dependent area of the articular cavity (ROI), it is possible to improve the visualization of the labrum and cartilage, avoiding artifacts (**B**). Some calcifications (arrow in (**C**)) can be clearly depicted in the same patients.

**Figure 3:**
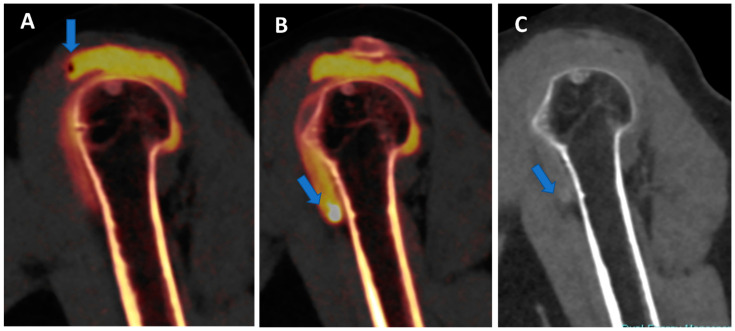
Potential pitfalls of DECTA. The sagittal reconstructed 1 mm iodine map from DECTA of the shoulder (**A**) shows an air bubble coming from injection in the sub-acromial space in a complete rotator cuff rupture (arrow). In the same patient (arrow on (**B**)), there is an ovoid dense image simulating a calcification in a dependent position. On sagittal high KeV VMI reconstruction (**C**), there is a complete subtraction of the area of highly concentrated contrast material (arrow).

Both shoulder and hip arthrography are used for preoperative evaluation in cases of dislocations, impingement, and dysplasia [4,5]. The assessment of morphology and anatomic angles is usually performed both on 2D and 3D reconstructions [19]. However, in the case of single-energy CT (SECT) arthrography, measurements could be invalidated because intra-articular iodinated contrast material overlays bone, especially as concerns 3D VRT reformats [19]. By using VNC imaging, DECTA allows the subtraction of iodine from the articular cavity, allowing for a reliable measurement of the glenoid or acetabular regions. In our experience, the subtraction can be performed during post-processing either by using a VMI application (by increasing the values of keV) or by obtaining VNC images from iodine maps (Figure 1 and Figure 2). The VMI application has the advantage that the values of KeV can be smoothly modulated to achieve the optimal visualization of contrast material for the purpose. For this reason, VMI should be the preferred application for reducing blooming artifacts in highly concentrated contrast material (Figure 3). This may represent an important pitfall of CTA because abnormally dense contrast could be visualized for several reasons in clinical practice, including peri-capsular extravasation during puncture, trapped contrast material in small intra-articular “recesses”, such as in operated patients with fibrous bands or synechiae, or simply because dense contrast material is accumulating in dependent regions.

In clinical practice, in cases of shoulder dislocation and bony glenoid lesions, CT represents the most reliable imaging tool for preoperative planning [37]. When shoulder instability becomes recurrent, surgery is indicated to fix the problem. Soft tissue damage includes anteroinferior labral detachment or capsular damage, leading to capsular redundancy. Bony instability is produced by lesions to the humeral head or the glenoid rim [37]. The risk of surgical repair failure increases as the radiologist fails to address the presence or correctly quantify the entity of a bone defect. Often, the reference standard for preoperative evaluation in patients with shoulder instability includes both an MRI to assess soft tissue injury and a non-contrast CT scan to assess bone damage. This may be problematic in that the additional non-contrast CT scan increases radiation exposure for the patient, and the MRI adds significant monetary cost and/or examination time.

Furthermore, there are two types of glenoid bone loss: fragment type and erosion type [38]. Both types are often associated with a Hill-Sachs lesion (HSL), representing a bone loss of the humeral head caused by the impact of the humeral head dislocated anteriorly with the glenoid rim. This is the case with the typical ‘bipolar lesion’. The contact zone between the glenoid rim and the humeral head is called the ‘glenoid track’. In the case of an HSL ‘on-track’ lesion, the risk of recurrent dislocation is low. Conversely, if HSL is ‘off-track’, the risk of engagement and dislocation is higher [38]. Once again, CT represents the most reliable imaging tool for the differentiation between ‘on-track/off-track’ lesions. For ‘off-track’ lesions, either remplissage or a Latarjet procedure is indicated, depending upon the glenoid defect size and the risk of recurrence [38].

In this scenario, DECTA may play a crucial role, allowing both soft tissue assessment and glenoid bony measurement to be performed on 2D and 3D virtual unenhanced images.

Moreover, imaging plays a critical role in the assessment of patients with femoroacetabular impingement [20]. MR arthrography is the best tool for imaging chondral and labral lesions [20]. However, the protocol should include a large-FOV fluid-sensitive sequence to exclude mimickers, radial imaging to determine the presence of a cam deformity, and imaging of the distal femoral condyles for measurement of femoral torsion. For this reason, the imaging protocol is usually relatively long. Conversely, CT has been considered a valuable tool for planning complex surgical corrections [20]. However, advanced imaging, such as 3D simulation, has the potential to improve surgical decision-making [20]. Although oblique axial MR images were initially used to describe the alpha angle, the extent of cam deformity could be underestimated because this dataset shows only the anterior position of the proximal femur [20]. Anatomic landmarks, such as the most prominent appearance of the greater trochanter (femoral 12-o’clock position) and the acetabular teardrop (acetabular 12-o’clock position), can be used for accurate topographic allocation of the cam deformity on radial images. A threshold of 60° has been introduced for an imaging diagnosis because it is associated with the progression of osteoarthritis within 2–5 years in patients with early symptoms. For clinical routine, morphologic and topographic assessment of osseous deformities of the femoral head and neck should be performed. Alpha angles are useful for identifying where the deformity is most pronounced [21]. Because of its wide availability, low cost, and high level of detail in assessing cortical and cancellous bone, CT remains an important modality in the diagnostic workup of FAI. Additionally, CT is still considered the imaging reference standard for measuring femoral torsion. Furthermore, CT-based 3D models of the pelvis are important for preoperative planning of complex osteotomies and can be used for surgical navigation [20].

Similarly to DECTA of the shoulder, DECTA of the hip could therefore represent a “one-stop one-shop” procedure that allows detailed assessment of chondral and labral defects, as well as 2D and 3D hip anatomy.

## 4. Iodine Maps and VNC Imaging

**Figure 4:**
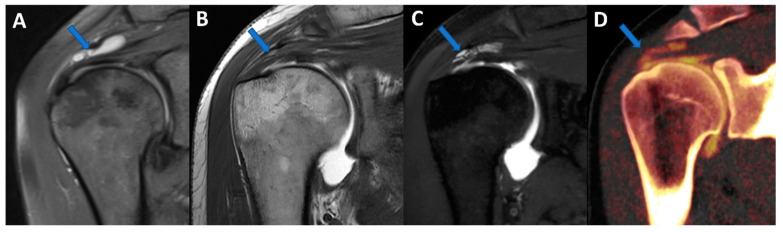
Complete supraspinatus tendon diagnosed using DECTA. On the coronal standard STIR MRI image (**A**), a fluid collection located on the bursal side of the supraspinatus tendon can be identified (arrow). On the coronal T1 weighted TSE MRA image (**B**), the supraspinatus tendon appears irregularly thinned, as in a case of a partial tear. There is no apparent passage of contrast material on the bursal side (arrow). On the corresponding 1 mm PD fat-saturated image of the coronal plane (**C**), there are still no clear signs of complete tendon tears (arrow). The reconstructed DECTA 1 mm coronal image (iodine map; (**D**)) clearly demonstrates the presence of a complete tear with the passage of contrast material on the bursal side of the tendon (arrow).

Iodine maps have been largely employed in body and vascular imaging to increase the contrast among soft tissues because iodine maps enhance the signal coming from iodine contrast [20,21,22,23]. In a recently published paper [5], DECTA of the shoulder was superior in the detection of glenoid labrum and rotator cuff tears with respect to standard CTA (sensitivity rose from 84.2% to 92.1% for reader 1; specificity rose from 77.8% to 88.9% for reader 2). Conversely, intra-observer agreement was higher for CTA if compared to DECTA [24]. In clinical practice, iodine maps can be used to enhance the visualization of tiny, subtle tears by modulating the vividness of contrast material, with a subsequent increase in contrast within soft tissues, including articular cartilages (Figure 4). The possibility of modulating the signal from injected contrast material may be very useful in cases of difficulty injecting contrast material in the articular cavity. For example, the signal can be augmented in cases of diluted or small amounts of injected contrast material or in cases with pre-existing intra-articular fluid. Conversely, the signal from injected contrast material could be reduced in the case of poorly diluted contrast material, causing artifacts that may obscure the adjacent bony or soft tissue structures. Additionally, in clinical practice, the signal coming from contrast material can be changed depending on the radiologist’s choice of non-destructive flow.

Additionally, DECT has been proposed for the identification of intra-articular loose bodies. In the study by Stern et al. [19], no difference was observed in the detection of periosteal calcifications between VNC and DECTA images (*p* = 0.29), while the detection of intra-articular loose bodies was superior on VNC images (*p* = 0.02). Moreover, the use of VNC images can improve confidence for both periosteal calcifications and intra-articular loose bodies (*p* < 0.001) [19].

**Figure 5:**
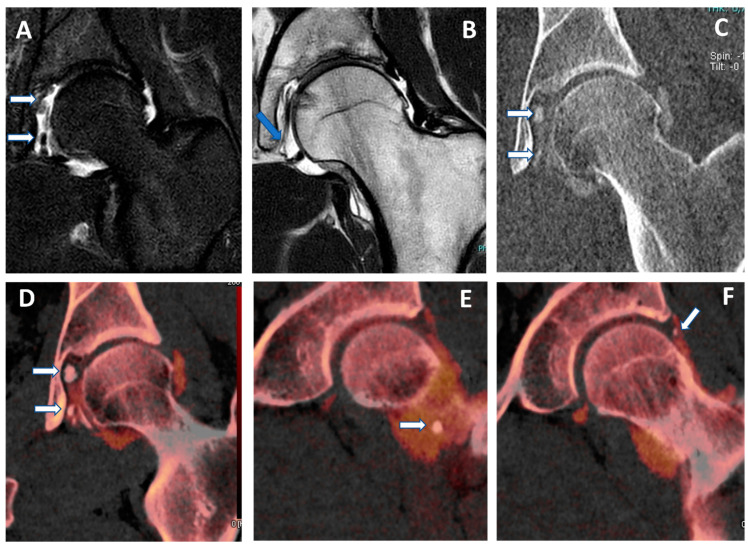
Capsular, labral, and loose calcifications in femoro-acetabular impingement. On MRA coronal STIR and T1 weighted images (**A**,**B**), it is possible to recognize subtle filling defects (arrows on (**A**)) and capsular thickening (blue arrow in (**B**)). On the DECTA VMI 1 mm coronal reconstructed image (**C**), calcifications are partially and erroneously subtracted (arrows). The corresponding DECTA iodine map images (**D**–**F**), reconstructed on the coronal plane (1 mm thickness), clearly show the presence of loose bodies and capsular and labral calcifications (arrows).

In our experience, both VMI and VNC images from iodine maps can be used to enhance the identification of loose bodies and to distinguish calcifications from non-calcified loose bodies. Moreover, these applications can be employed, alone or in combination, to avoid potential pitfalls, such as tendon or labral calcifications (that may mimic the passage of contrast material in the case of a subtle tear) or the abnormal accumulation of highly concentrated contrast material in a distal articular recess, simulating the presence of intra-articular calcifications (Figure 5).

**Figure 6:**
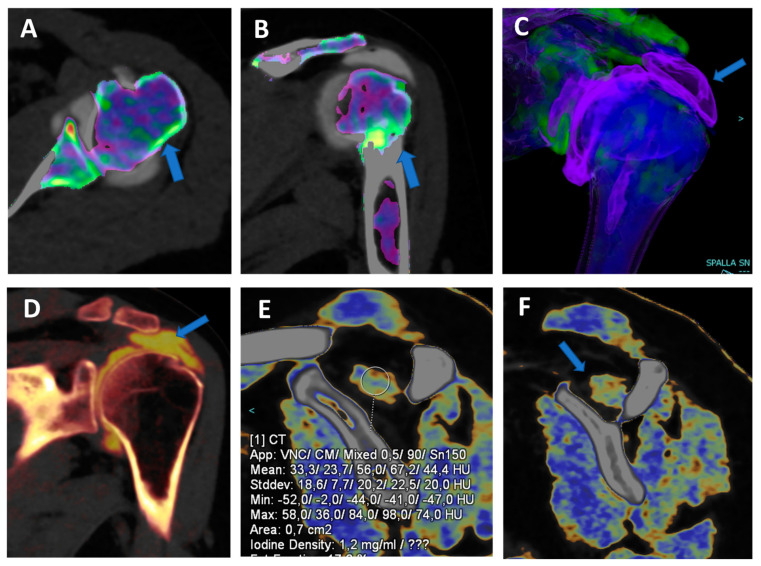
Traumatic anterior shoulder dislocation with rotator cuff complete tear and Hill Sachs lesion. On the 2D 1 mm axial and coronal reconstructed VNCa images (**A**,**B**), a subtle depression of the posterior aspect of the humeral head is visible, with mild edema coded in green on the superimposed map (arrow). The 3D VNCa image (**C**) clearly shows the passage of contrast material in the sub-acromial space (arrow). On the coronal 1 mm reconstructed iodine map image (**D**), the complete rupture of the rotator cuff is beautifully confirmed (arrow). On the sagittal LNC images (**E**,**F**), it is possible to identify (arrow on (**F**)) and quantify (ROI on (**E**)) the presence of atrophy of the muscle belly.

VNC images can generate a “fat map” that could be used to identify and quantify the presence of atrophy of the peri-articular muscle belly (Figure 6) [25]. In the study by Molwitz et al., dual-energy CT material decomposition and virtual non-contrast-enhanced DECT HU values were successfully employed to reliably assess muscle fat [25]. In particular, studying 21 patients and using MRI as the reference for diagnosis, the authors measured HU values on VNC DECT images in 126 regions of interest within the posterior paraspinal muscle, achieving a very good correlation between DECT and MRI (r = 0.91; r = −0.98). For this reason, dual-energy computed tomography could represent an alternative tool for the assessment of muscle quality, an important parameter for the risk of recurrent tendon or ligament tears around joints.

## 5. Virtual Non-Calcium (VNCa) Imaging

DECT has been successfully used to identify BME in traumatic and non-traumatic settings [14,15], with high diagnostic accuracy values both for spine and appendicular skeletal imaging [26,27,28,29,30]. The BME detection rate is higher in cases with severe edema in comparison to patients with mild edema and tends to increase in elderly patients because of the relative increase in yellow marrow and thinning of cortical bone [28]. Although specificity could be low, especially in the case of young patients with thick cortical bone or peri-articular bone sclerosis, DECT could play a role when MRI availability is limited or an MRI is contraindicated [28].

**Figure 7:**
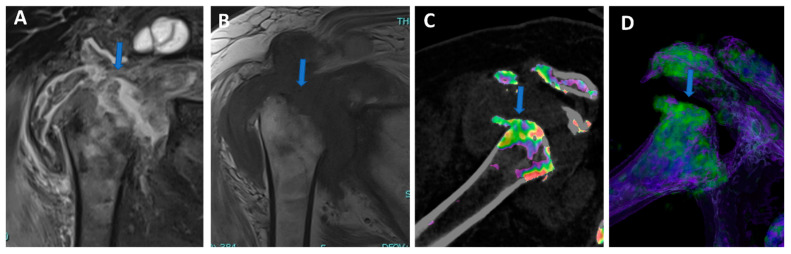
Shoulder acute crystal arthritis. On coronal STIR and T1 weighted MR images (**A**,**B**), it is possible to identify erosive changes and BME of the humeral head (arrow) with corpuscular fluid within the articular cavity. On para-coronal 2D and 3D DECT images (**C**,**D**), severe bone reabsorption with edema of the femoral head is confirmed (arrow).

In clinical practice, BME maps reconstructed from VNCa imaging could be employed to detect bone marrow lesions around the imaged joints. Although iodinated contrast material could generate some artifacts in the adjacent areas, the presence of BME can still be visualized. For example, in the case of shoulder trauma with doubtful dislocation, the presence of BME on the posterior aspect of the humeral head may help corroborate the diagnosis of a Hill-Sachs lesion (Figure 1 and Figure 6). In addition, BME around the joint, with or without erosions, can be found in inflammatory diseases such as septic or aseptic arthritis [31,32,33] (Figure 7).

In particular, DECT has recently been proposed for the diagnosis of osteomyelitis of the lower limb [34]. Osteomyelitis is an infection of the bone that involves the medullary canal but may also involve adjacent joints. In the study, 44 patients were enrolled, with 32 positive cases. DECT achieved an overall AUC of 0.88 and an AUC of 0.85 as regards the detection of BME. However, the diagnosis of infection was also based on the presence of bone erosions, which were better identified with DECT compared to MRI (*p* = 0.02).

## 6. High-Resolution, Isotropic CT Images

**Figure 8:**
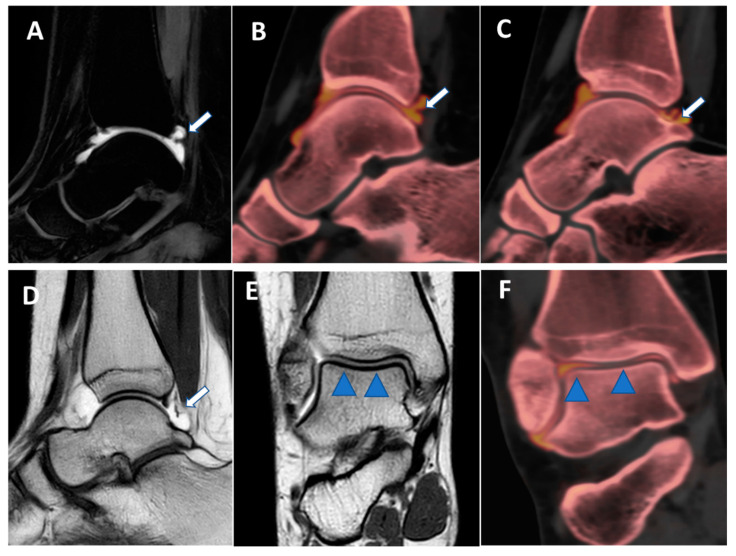
Posterior capsular plica at ankle arthrograms. On 3 mm sagittal STIR and T1 weighted images (**A**,**D**), a thin posterior plica (arrow) is depicted. The same plica is nicely depicted on the corresponding sagittal 0.3 mm DECT iodine map images (arrows on (**B**,**C**)). On 3 mm Coronal T1 weighted images (**E**), a partial volume effect may hinder subtle cartilage defects (blue arrowheads). On the corresponding 0.3 mm coronal DECTA (**F**), articular cartilages are optimally imaged without any artifacts.

**Figure 9:**
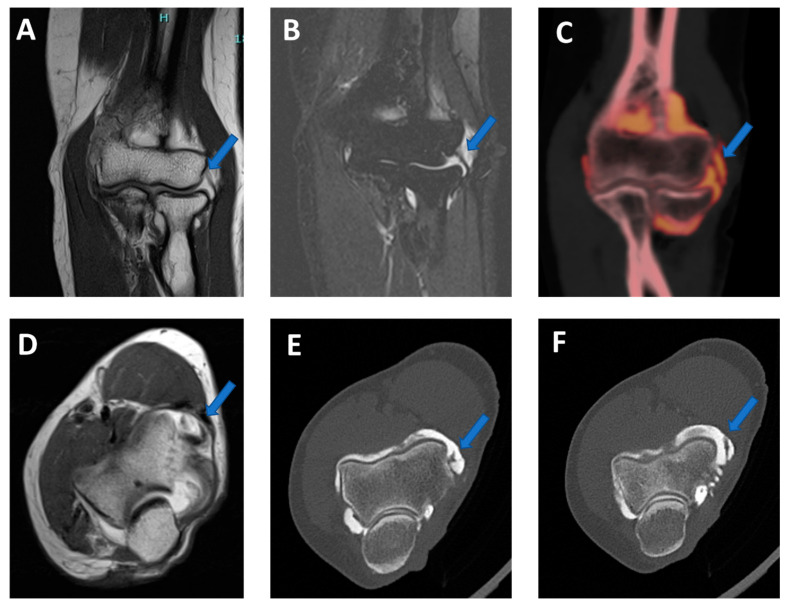
Lateral plica syndrome at elbow arthrography. On the 3 mm coronal STIR and T1 weighted images (**A**,**B**), a tiny lateral plica (arrow) is depicted. The same plica is nicely depicted (arrow) on the corresponding reconstructed coronal 0.5 mm DECT iodine map image (**C**). On the 3 mm axial T1 weighted image (**D**), a partial volume effect does not allow for the correct image of the plica (arrow) and articular cartilages. On the corresponding 0.5 mm axial DECTA (**E**,**F**), the plica (arrow) and the adjacent articular cartilages are beautifully visualized without any artifact.

**Figure 10:**
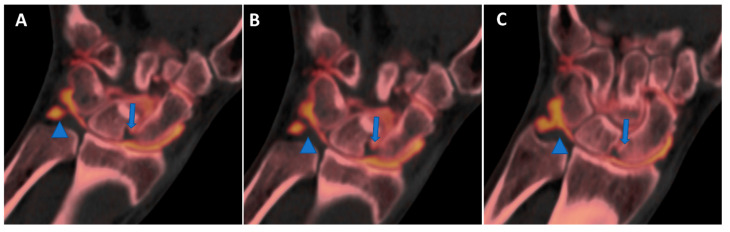
High-spatial-resolution wrist anatomy using DECTA. On the 0.4 mm coronal DECTA iodine map images (**A**–**C**), a triangular fibrocartilage complex (arrowhead) and a scapho-lunate ligament (arrow) are nicely depicted.

One of the major intrinsic advantages of CT is the possibility of achieving high spatial resolution images with bone or soft-tissue windows [39]. In clinical practice, recent DECT scanners allow for 0.3 mm isotropic images that can be reconstructed on any imaging plane, simulating rotational images acquired on MRI and correctly evaluating complex anatomic structures. Additionally, thanks to the additional advantage of the intrinsic high contrast resolution of DECT, these images can help with the visualization of cartilages, intra-articular plicae, and the normal anatomy of tiny anatomic structures in small joints of the wrist (Figure 8, Figure 9 and Figure 10).

## 7. Tendons and Ligaments

DECT’s intrinsic high contrast can be used for the evaluation of collagenous structures. Using collagen postprocessing techniques may increase diagnostic confidence in assessing disc bulging, tendons, and ligament tears [9]. DECT is less sensitive than MRI in the evaluation of small lesions of tendons and ligaments, but by injecting contrast material, it is possible to increase the visibility of lesions with similar results to MRI. For example, in the shoulder, the passage of contrast material in sub-acromial space, clearly visible using DECT, is a direct sign of an RC tear.

In subjects with subacute to chronic traumatic ACL disruption confirmed by MR imaging, receiver operating characteristic curve analysis of DECT performance showed that sagittal oblique images with dual-energy bone removal, soft tissue windowing, and single-energy bone removal were most accurate, with area under the curve (AUC) of 0.95, 0.94, and 0.93, respectively [35]. DECTA has shown high diagnostic accuracy values in depicting glenoid labral and rotator cuff tears in the shoulder [5]. DECT is particularly well suited to the assessment of traumatic joint injuries. There is less beam hardening artifact in the knees and ankles when compared to the shoulders or pelvis, and the marrow spaces are large when compared to the smaller peripheral bones [36]. Additionally, the typical pattern of BME around the joints can assist the radiologist in determining the integrity of the ligaments, whose assessment is enhanced by collagen imaging [36]. Visualization of the collateral ligaments and menisci is also enhanced using DECT collagen mapping techniques. Collagen mapping is also applicable to other joints, including the wrists and ankles. This is particularly useful in the assessment of displaced fractures, where tendon entrapment will alter orthopedic surgical management [36].

However, to the best of our knowledge, there are no data available on human subjects regarding the accuracy of DECT arthrography in depicting tendon or ligament tears in other joints.

## 8. Metallic Artifact Reduction

DECT represents a powerful tool for the evaluation of hip and knee prosthesis loosening in clinical practice [16,24]. In the knee, as concerns tibial analysis, DECT performed better than SECT (mean sensitivity and specificity for arthroplasty loosening were 88% and 91%, versus 73% and 78%, respectively). Additionally, the overall diagnostic performance of DECT (AUC, 0.87) for the femur was superior to both SECT and radiography (*p* = 0.001) [24].

Similarly, in the hip, dual-energy CT showed better diagnostic performance than conventional radiography (CR) in diagnosing hip prosthesis loosening, showing higher sensitivity and specificity than CR (94% and 95% versus 84% and 91%) [16].

**Figure 11:**
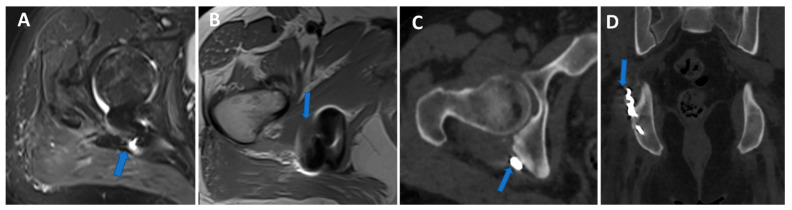
Persistent hip pain in previous trauma with a surgically treated pelvic fracture. Axial 3 mm STIR and T1 weighted images (**A**,**B**) show metallic-induced artifacts around the posterior ischial tuberosity (arrow). On axial and coronal 1 mm VMI reconstructed images (**C**,**D**), it is possible to control artifacts and depict the presence of metallic hardware projecting on the course of a thickened right sciatic nerve (arrow).

By reducing peri-prosthetic artifacts by using the VMI application, DECT improves the visualization of the bone-prosthesis interface, helping to identify loosening and other phenomena [16,17,18,19,20,21,22,23,24]. In this scenario, VMI can be used to reduce the artifacts around peri-articular hardware as well (Figure 11).

Although DECT allows for a reduction in metal-hardware artifacts, it cannot help in the identification of concomitant BME around the prostheses because it is obscured by the artifacts. It can be used in combination with other software to bypass this problem, but it is not frequently used.

## 9. Conclusions

Arthrography represents a complex exam during clinical practice, often employed for pre-operative assessment. MRA represents the most reliable imaging tool because of its intrinsically high contrast resolution for soft tissue imaging. Standard CTA represents a valid alternative exam, being a fast and cheap exam with high spatial resolution concerning bone imaging. However, several DECT applications can be used in clinical practice to increase the contrast in soft tissue and reduce the gap with MRA. In this setting, CT arthrograms should always be acquired and reconstructed in dual-energy mode, allowing for improved CTA diagnostic accuracy.

## Data Availability

No additional data available for this review study.
